# *Xenopus* Reduced Folate Carrier Regulates Neural Crest Development Epigenetically

**DOI:** 10.1371/journal.pone.0027198

**Published:** 2011-11-09

**Authors:** Jiejing Li, Yu Shi, Jian Sun, Yanfeng Zhang, Bingyu Mao

**Affiliations:** 1 State Key Laboratory of Genetic Resources and Evolution, Kunming Institute of Zoology, Chinese Academy of Sciences, Kunming, China; 2 Graduate University of Chinese Academy of Sciences, Beijing, China; University of Insubria, Italy

## Abstract

Folic acid deficiency during pregnancy causes birth neurocristopathic malformations resulting from aberrant development of neural crest cells. The Reduced folate carrier (RFC) is a membrane-bound receptor for facilitating transfer of reduced folate into the cells. RFC knockout mice are embryonic lethal and develop multiple malformations, including neurocristopathies. Here we show that *XRFC* is specifically expressed in neural crest tissues in *Xenopus* embryos and knockdown of *XRFC* by specific morpholino results in severe neurocristopathies. Inhibition of RFC blocked the expression of a series of neural crest marker genes while overexpression of RFC or injection of 5-methyltetrahydrofolate expanded the neural crest territories. In animal cap assays, knockdown of RFC dramatically reduced the mono- and trimethyl-Histone3-K4 levels and co-injection of the lysine methyltransferase hMLL1 largely rescued the *XRFC* morpholino phenotype. Our data revealed that the RFC mediated folate metabolic pathway likely potentiates neural crest gene expression through epigenetic modifications.

## Introduction

Neural crest (NC) is a multipotent cell population which originates at the border between the neural plate and epidermis during vertebrate development. Later on, these cells migrate to various places throughout the body and give rise to various tissues, including craniofacial bones and cartilages, melanocytes, cardiac structures and peripheral nervous system [1]. Defective neural crest development leads to a broad spectrum of congenital malformations, collectively called neurocristopathies, which includes defects in pigmentation, and abnormal craniofacial and heart development. Several neurocristopathies have been documented, including frontonasal dysplasia, Waardenburg-Shah syndrome, DiGeorge syndrome, CHARGE syndrome, congenital nevi, and Hirchsprung disease [2], [3].

Induction, specification, migration, and differentiation of the NC cells are tightly regulated by a carefully orchestrated multi-step gene regulatory network (GRN) [1], [4]–[6]. Neural crest formation occurs in a series of tightly regulated steps. First, the presumptive neural crest territory is induced at the dorsal neural plate border through the interplay of different signaling pathways including BMPs, Wnts and FGFs. These signals control the broad expression of a set of transcription factors at the neural plate border region, including Pax3, Msx1 and Zic1. These neural plate border specifiers further turn on the expression of a group of genes in the emerging neural crest cells, including Snail1, Snail2, FoxD3, Sox10, Sox9, and Twist1. These neural crest genes are extensively cross-regulated and many of them have been shown to be necessary and/or sufficient for the expression of many other genes. The neural crest specifier genes further control the expression of several downstream mediators of neural crest migration. Terminal differentiation of the neural crest cells is regulated by different networks.

Folate deficiency has long been known to contribute to developmental neural defects, especially neural tube defects and neurocristopathies [7]–[10]. In humans, it has been documentated folic acid prevents the development of neural tube defects, craniofacial malformation, and heart defects [11]–[13]. In addition, *in vivo* and *in vitro* experiments suggest that altering levels of folic acid leads to aberrant cardiac NC cell migration and differentiation in chick [14], [15]. Folate is a cofactor in one-carbon metabolism and is a crucial regulator of nucleotide synthesis and methylation reactions. 5-methyltetrahydrofolate (5-MTHF) is involved in the remethylation of homocysteine to methionine, which is the precursor of S-adenosylmethionine (SAM), the primary methyl group donor for most biological methylation reactions [8]. In humans, folate metabolism and folate status has been shown to affect the global methylation of DNA [16].

As a water-soluble B class vitamin, the uptake of folate by cells is mediated by specific carriers or receptors, including folic acid receptors (FRs), proton-coupled folic acid transporter (PCFT), and reduced folic acid carrier (RFC). RFC, a 12 transmembrane protein, is believed to be the major transporter for 5-MTHF, which is the major form of folate in circulation [17]. RFC has a low affinity for folic acid and a high affinity for reduced folate and methotrexate (MTX), an antifolic acid chemotherapeutic drug [18]. RFC is widely expressed in various human tissues and mouse embryos [19], [20]. *RFC1* knockout mice die shortly after implantation. Supplementation of high dose of maternal folate prolongs the survival of *RFC1* null embryos until mid-gestation, but these embryos develop multiple malformations, including defects in the neural tube, craniofacial and cardiac malformations [21]. Such malformations, including the craniofacial and heart defects, coincide with neurocristopathies [3], suggesting that RFC might be required for normal neural crest development [7].

Here we show in *Xenopus* embryos that RFC was involved in neural crest development and knockdown of *XRFC* by specific morpholino led to severe neurocristopathies as observed in mice. We also provide evidence that the folate-mediated metabolism regulates neural crest gene expression is likely through epigenetic mechanisms.

## Results

### Xenoups RFC is expressed in the neural crest territories

**Figure 1:**
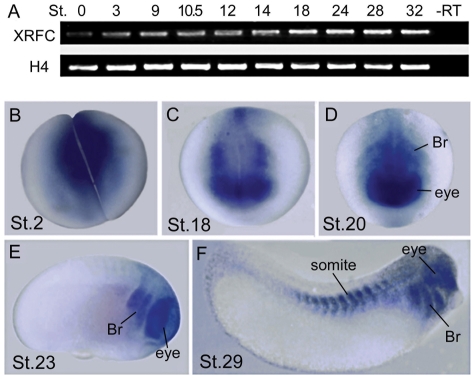
Temporal and spatial expression pattern of *XRFC*. (A) *XRFC* is expressed maternally and throughout the stages examined (St.0 to St.32) as detected by RT-PCR. –RT, negative control without reverse transcriptase in the RT reaction. (B–F) Expression of *XRFC* at the indicated stages revealed by *in situ* hybridization. (B) The maternal XRFC expression is detected at the animal pole at 2-cell stage. (C) At stage 18, *XFRC* is expressed at the anterior neural plate and its border. (D–E) At the late neurula stages, the expression of *XRFC* is detected in the prospective eye and forming branchial arches. (F) At tailbud stage, *XRFC* is strongly expressed in the branchial arches, eyes, head mesenchyme and the somites. Br, branchial arch.

We examined the temporal and spatial expression pattern of *XRFC* during early *Xenopus* embryogenesis by RT-PCR and whole-mount *in situ* hybridization. *XRFC* was expressed in *Xenopus* embryos throughout the stages examined from St.0 to St.32 (Fig. 1A). Maternal *XRFC* transcripts were localized to the animal pole at 2-cell stage (Fig. 1B). At stage 18, *XRFC* was expressed at the anterior neural plate and its border (Fig. 1C). At late neurula stages (Fig. 1D, E), the expression of *XRFC* was detected in the prospective brain, optic vesicles, and the developing branchial arches. During the tailbud stages, *XRFC* expression remained high in the branchial arches, eyes, head mesenchyme and the somite regions (Fig. 1F). The expression pattern of *XRFC* overlaps with the neural crest territories, including the neural plate border, branchial arches, somites and the head mescenchyme. This suggests RFC may play a role in neural crest development.

### XRFC is required for neural crest development

**Figure 2:**
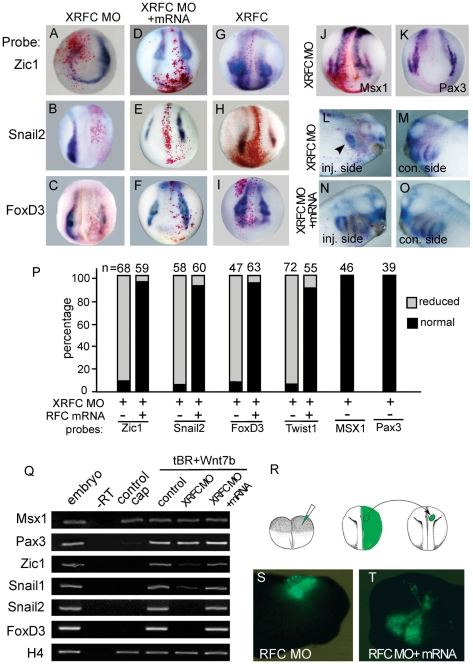
*XRFC* is required for neural crest induction and migration. (A–K) XRFC-MO and tracing lacZ mRNA were injected into one cell of four-cell stage embryos, the expression of neural crest makers were examined at stages 15–17. (A–C) Knockdown of XRFC by morphoino inhibits the expression of *Zic1*, *Snail2* and *FoxD3* in the injected sides (labeled red by staining of the tracing lacZ). (D–F) Co-injection of *XRFC* mRNA (800 pg) rescues the expression of *Zic1*, *Snail2* and *FoxD3*. (G–I) Overexpression of *XRFC* expands the expression domains of *Zic1*, *Snail2* and *FoxD3*. (J, K) XRFC morpholino injection does not affect the expression of *Msx1* and *Pax3.* (L–O) *Twist1* staining of neural crest cells at stage 32 of embryos injected on one side with XRFC-MO or XRFC-MO plus rescue mRNA. In XRFC-MO injected side, the neural crest cells are retained in a dorsal position (arrowhead in L). (P) Quantification of the effect of XRFC-MO on the expression of neural crest markers and the rescue by RFC mRNA. (Q) RT-PCR analysis in animal cap assay to show the effect of XRFC-MO on the expression of the indicated neural crest genes induced by co-injection of *tBR* and *XWnt7b*. (R–T) Transplantation experiment to show the effect of XRFC-MO on neural crest migration. (R) Schematic drawing showing the transplantation procedure. (S) The EGFP-labeled XRFC-MO injected neural crest tissue fails to migrate at tailbud stage after transplantation. (T) Co-injection of *XRFC* mRNA restores the migration ability of the neural crest cells. inj. side, injected side; con. side, control side.

We further examined whether XRFC knockdown would affect neural crest development. XRFC-MO, together with *lacZ* mRNA as a lineage tracer, were injected into one blastomere of the embryos at four cell stage, such that the contralateral uninjected sides serve as an internal control. At the neurula stage, expression of neural crest maker genes *Zic1*, *Snail2*, and *FoxD3* were clearly down-regulated in the injected side (Fig. 2 A–C, P) compared to the control side. This effect could be largely rescued by co-injection of a morpholino resistant *XRFC* mRNA (Fig. 2 D–F, P). Interestingly, over-expression of *XRFC* mRNA alone could promote the expression of *Zic1* (92%, n = 37), *Snail2* (96%, n = 47), and *FoxD3* (95%, n = 41; Fig. 2 G–I). In contrast, the expression of *Msx1* and *Pax3* was not affected by XRFC-MO or *XRFC* mRNA injections (Fig. 2 J, K, P and data not shown).

We also tested whether XRFC is required for neural crest induction in animal cap assays. Neural crest cells can be induced in animal caps by coinjection of Wnts and neuralizing factors [22]. Animal caps were dissected at stage 9 from embryos injected with *XWnt7b* and *tBR* mRNA, with or without XRFC-MO. We first confirmed that XRFC was expressed in control and induced animal caps (data not shown). As in whole embryos, suppression of XRFC significantly reduced the expression of *Zic1*, *Snail2* and *FoxD3* without significantly affecting *Msx1* and *Pax3* expressions (Fig. 2 Q).

We checked whether RFC knockdown also affects neural crest migration using *Twist1* as a marker. At the taibud stage, *Twist1* labeled NC cells migrate ventrally in several streams into the hyoid and branchial arches. In the XRFC*-*MO injected embryos, however, *Twist1* positive NC cells failed to migrate ventrally into separate streams, but were clustered and retained in a dorsal position (Fig. 2 L, M, P). This effect could be rescued by co-injection of the *XRFC* mRNA (Fig. 2 N, O, P). We next examined the effects of XRFC-MO on neural crest migration using a transplantation experiment ([23], Fig. 2 R). When the presumptive neural crest tissue from an embryo injected with EGFP mRNA is transplanted into similar place of a normal embryo at the neurula stage, these cells will migrate ventrally into the branchial arch regions at tailbud stage. When tissues from the XRFC-MO injected embryos were transplanted, however, these cells stayed at the transplanted position and failed to migrate ventrally (100%, n = 14, Fig. 2 S). This defect could be rescued by co-injection of *RFC* mRNA (100%, n = 15, Fig. 2 T). We can not rule out the possibility that the failure of neural crest migration in XRFC-MO injected embryos might be a secondary effect of the defected neural crest differentiation.

### RFC knockdown leads to neurocristopathic malformations in Xenopus embryos

**Figure 3:**
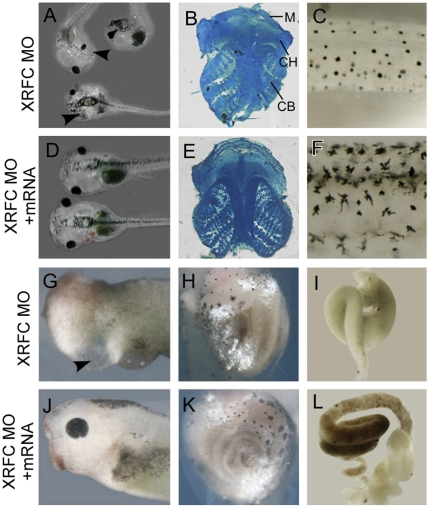
Knockdown of XRFC leads to multiple malformations at tadpole stages. (A) Knockdown of XRFC caused craniofacial malformations (arrowheads) with curled trunks. (B) XRFC-MO inhibited the formation of cranial cartilage. Cartilages in late tadpoles were stained with alcian blue. Cartilage on the RFC-MO injected side was often malformed and reduced. M, meckel's cartilage; CH, ceratohyal cartilage; CB, ceratobranchial cartilage. (C) The XRFC-MO injected embryos were hypopigmented at tadpole stage. (G) The XRFC morphants showed enlarged pericardial cavity (arrowhead), indicative of heart defects. (H–I) The gut coiling in XRFC was malformed at tailbud (H) and tadpole (I) stages compared to the rescued group (K, L). (D, E, F, G, K, L) Co-injection of XRFC mRNA rescued the above mentioned malformations.

We checked whether knockdown of XRFC could induce neurocristopathic phenotypes at later stages. At tadpole stages, the XRFC-MO injected embryos develop craniofacial malformation with curled trunks (91%, n = 93; Fig. 3 A). The cranial cartilages were atrophied (Fig. 3 B). Also the embryos were hypopigmented and developed heart defects (as indicated by the enlarged pericardial cavity) and gut coiling malformations (Fig. 3 C, G, H, I). These defects could be rescued by co-injection of *XRFC* mRNA (94%, n = 86; Fig. 3 D, E, F, J, K, L). Thus knockdown of XRFC in *Xenopus* embryos reproduces the neurocristopathic phenotypes as observed in mice with deleted *RFC* [21], further confirming that RFC is required for neural crest development in *Xenopus*.

### RFC is involved in the neural crest regulatory network

**Figure 4:**
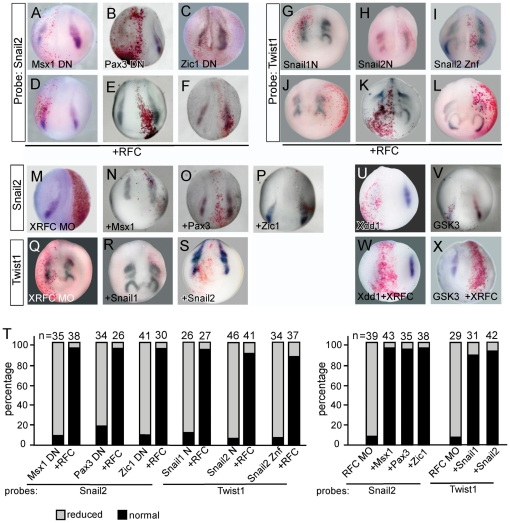
RFC is involved in the neural crest gene regulatory network. (A–F) The inhibition of Snail2 expression by dominant negative Msx1, Pax3 and Zic1 was rescued by co-injection of XRFC. (G–L) Snail1 N, Snail2 N and Snail2 Znf inhibited the migration of neural crest (as indicated by the distribution of the *Twist1* positive neural crest cells, G–I), which was rescued by co-injection of *XRFC* (J–L). (M–P) The inhibition of Snail2 expression by XRFC-MO was rescued by Msx1, Pax3 and Zic1 co-expression. (Q–S) The neural crest migration defects in XRFC morphants were rescued by co-injection of *Snail1* or *Snail2*. (T) Quantification of the rescue effect of RFC on the expression of neural crest markers inhibited by dominant negative Msx1, Pax3, Msx1, Snail1, and Snail2 (left panel) and the rescue of the XRFC-MO effect by Msx1, Pax3, Zic1, Snail1, and Snail2 (right panel). (U–X) Dominant negative dishevelled (Xdd1) and GSK3β blocked the expression of *Snail2* (U, V), which could not be rescued by *XRFC* (W, X).

Our knockdown data suggest that RFC is required for the proper expression of a series of neural crest genes and overexpression of RFC promotes their expression. To test whether XRFC could be fit into the NC GRN linearly, we carried out a series of knockdown and rescue experiments. The neural border specifiers Pax3, Msx1 and Zic1 are at an upstream position of the NC GRN hierarchy. Interfering with their function using dominant negative constructs inhibited neural crest induction (Fig. 4 A–C, T, and Fig. S1). When XRFC was co-expressed, however, the dominant negative effects of all these three constructs were rescued (Fig. 4 D–F, T). On the other hand, Pax3, Msx1 and Zic1 could also rescue the neural crest defects in the XRFC-MO injected embryos (Fig. 4 M–P, T, and Fig. S1). Similar situation holds true for more downstream genes *Snail1* and *Snail2*. XRFC rescued the inhibitory effect of the dominant negative *Snail1* and *Snail2* constructs on neural crest gene expression (Fig. 4 G–I, T and Fig. S1). Furthermore Snail1 and Snail2 also rescued the expression of the neural crest markers in the XRFC morphants (Fig. 4Q–S, T and Fig. S1). Since RFC has no direct effect on *Pax3* and *Msx1* expression, these data suggest that RFC might have a general role in potentiating the expression of *Zic1* and downstream genes.

In addition to its involvement in early induction stage, Wnt signaling is also required for the activation of neural crest genes by Pax3 and Zic1 [6]. Blocking of Wnt signaling by dominant negative *Dishevelled (Xdd1)* [24] or *GSK3β* abolished neural crest induction (88%, n = 44 and 85%, n = 55 respecitvely, Fig. 4 U, V). Co-injection of XRFC mRNA failed to rescue the neural crest defects (94%, n = 41 and 95%, n = 51 respectively, Fig. 4 W, X), suggesting a stringent requirement of Wnt signaling in neural crest development.

### XRFC regulates neural crest development epigenetically

**Figure 5:**
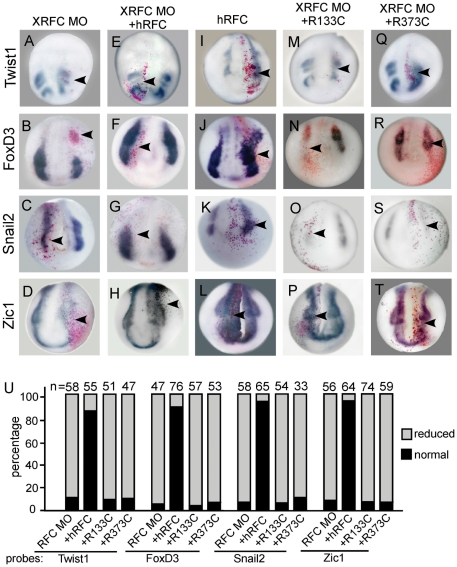
The effects of different hRFC constructs on neural crest gene expression. (A–H) XRFC-MO injection blocked neural crest maker expression which was rescued by the wild type hRFC. (I–L) Overexpression of hRFC promoted of the expression of neural crest genes. (M–T) The two mutated form of hRFC (R133C, R373C) failed to rescue the neural crest gene expression in the XRFC morphants. The arrowheads indicate the injected sides. (U) Quantification of the rescue of the XRFC-MO effect by the wild type and mutated hRFC constructs.

We tested whether the role of XRFC in neural crest development is associated with its function as a folate carrier. We used two loss-of-function hRFC (R133C and R373C) mutants [25], which were known to be defective in folate transportation. Co-injection of wild type *hRFC* rescued the effect of XRFC-MO on neural crest gene expression (Fig. 5A–H, U ) and over-expression of *hRFC* promoted neural crest gene expression (Fig. 5 I–L, Fig. S2). The two hRFC mutants, however, failed to rescue the XRFC knockdown phenotype (Fig. 5 M–T, U) or to promote the expression of neural crest markers (Fig. S2).

**Figure 6:**
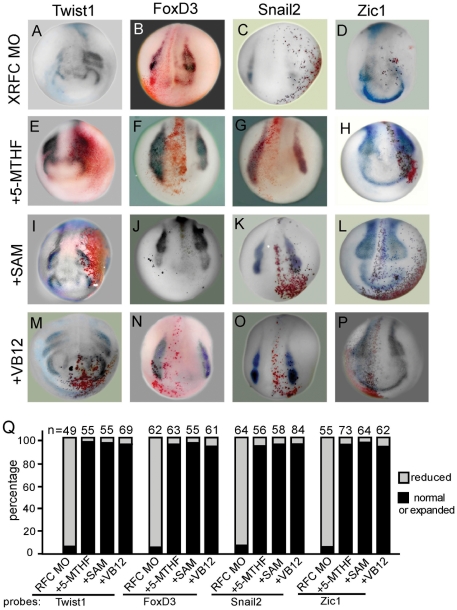
XRFC regulates neural crest gene expression through the one-carbon metabolism pathway. Co-injection of 5-MTHF (20 ng/embryo, E–H), SAM (10 ng/embryo, I–L) or Vitamin B12 (50 ng/embryo, M–P) with XRFC-MO rescued the expression of the neural crest maker genes (compare with A–D). (Q) Quantification of the rescue of the XRFC-MO effect by 5-MTHF, SAM, and Vitamin B12.

5-methyltetrahydrofolate (5-MTHF) is the major form of folate transported by RFC. We tested whether 5-MTHF itself could rescue the XRFC knockdown phenotype. Indeed, co-injection of 5-MTHF clearly rescued XRFC-MO-induced neural crest defects (Fig. 6 A–H, Q). Injection of 5-MTHF also promoted neural crest development as indicated by the expansion of the neural crest markers (Fig. S2). On the other hand, MTX, an antagonist of folic acid, disrupted neural crest induction on the injected side, which can further be rescued by 5-MTHF (Fig. S3).

We then tested whether RFC regulated neural crest development by promoting the methylation cycle. In the cell, the methyl group of 5-MTHF is transferred to homocysteine, via vitamin B12, to generate methionine, which is the substrate for the synthesis of S-adenosylmethionine (SAM). SAM is a direct donor of the methyl group in methylation of protein and DNA. Co-injection of SAM or vitamin B12 with XRFC-MO both rescued the expression of *Zic1*, *Snail2*, *FoxD3,* and *Twist1* (Fig. 6 I–Q). Furthermore, injection of SAM or vitamin B12 also promoted the expression of the neural crest markers (*Zic1*, *Snail2*, *FoxD3* and *Twist1*) (Fig. S2). These results suggest that RFC likely promotes neural crest development through the methylation cycle.

**Figure 7:**
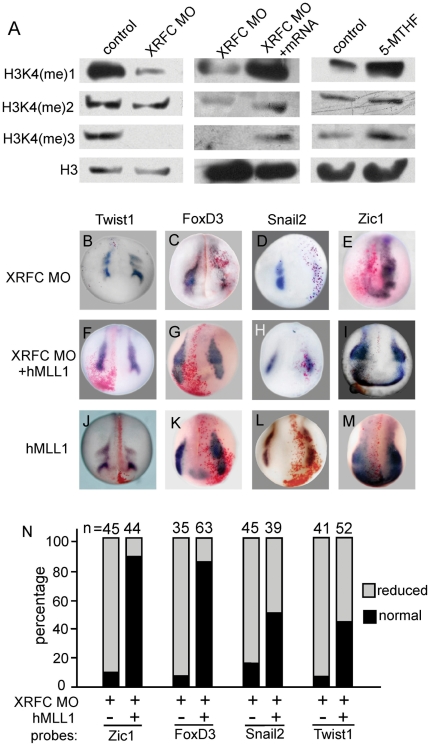
RFC regulated neural crest early development through histone modification. (A) Levels of different methylation forms of histone 3 in animal caps injected with *XWnt7b* and *tBR* with or without XRFC-MO. Knockdown of XRFC by injection XRFC-MO decreased both the mono- and trimethyl-H3-K4 levels but not dimethyl-H3-K4 level (left panel), which was restored by co-injection of *XRFC* mRNA (middle panel). Injection of 5-MTHF increased the mono- and trimethyl-H3-K4 levels but not dimethyl-H3-K4 level (right panel). (B–I) *hMLL1* plasmid co-injection rescued the effect of XRFC-MO on the expression of Zic1 and FoxD3, and weakly on Snail2 and Twist1. (J-M) Overexpression of hMLL1 alone promoted the expression of Zic1 and FoxD3, but had no clear effects on Snail2 and Twist1. (N) Number of embryos showed reduced expression of *Zic1*, *FoxD3*, *Snail2* and *Twist1* injected with XRFC-MO with or without *hMLL1* plasmid (50 pg/embryo).

The methylation cycle may regulate gene expression via epigenetic regulations, i.e., promoting DNA and histone methylation. As DNA methylation mainly results in gene inactivation and silencing [26], we checked whether knockdown of RFC affected histone methylation levels. High levels of H3K4 trimethylation have been shown to be associated with the 5′ regions active genes [27]. We failed to detect apparent changes of H3K4 methylation levels in whole embryos injected with XRFC-MO or mRNA (data not shown), probably due to the restricted expression of endogenous XRFC and high levels of background histone methylation. We then tested the H3K4 methylation levels in the neural crest induction assay in animal caps injected with *XWnt7b* and *tBR* with or without XRFC-MO. In this system, knockdown of XRFC clearly reduced the mono- and trimethyl-H3-K4 levels (Fig. 7A) which was restored by co-injection of *XRFC* mRNA. Injection of 5-MTHF also increased both the mono- and trimethyl-H3-K4 levels, but not dimethyl-H3-K4 level. These data suggest that endogenous RFC is required for proper histone methylation regulation *in vivo*.

### The expression of neural crest genes are subjected to epigenetic regulation

As a marker for gene activation, the methylated H3K4 (especially H3K4me3) is predominantly enriched surrounding transcriptional start sites [27]. We checked whether the neural crest genes are potentially regulated by histone3 methylation using published H3K4me3 chromatin immunoprecipitation data. Based on bioinformatics analyses of published genome-wide H3K4me3 pattern of *Xenopus* [28], zebrafish embryos, and human and mouse embryonic stem cells, H3K4me3 modification were highly enriched in the flanking and coding regions of *FoxD3*, *Zic1*, and *Twist1* in all species and sporadically enriched in *Snail2* (data not shown), indicating functional constraints of epigenetic regulation of these genes.

The H3K4 methylation is mediated by a group of lysine methyltransferases [29]. Among them, the *MLL* subfamily genes appear to play important roles in early development. Deletion of *MLL1-3* genes in mouse are all lethal [30]–[32]. *MLL1* knockout mice also show defects in neural crest derivatives, e.g., branchial arches[33]. We tested whether *MLL1* could rescue the neural crest defects in XRFC-MO injected embryos. We used hMLL1 [34] plasmid injection in our rescue experiments. Interestingly, overexpression of *hMLL1* was sufficient to rescue the expression of *Zic1* and *FoxD3* in XRFC-MO injected embryos (Fig. 7F–I). In contrast, the expression of *Snail2* and *Twist1* were only partially rescued (Fig. 7F–I, N), even using higher doses of *hMLL1* (data not shown). In addition, overexpression of hMLL1 alone promoted the expression of *Zic1* and *FoxD3* (81%, n = 44 and 93%, n = 45 respectively, Fig. 7 K, M), but not *Snail2* and *Twist1* (0%, n = 39 and 0%, n = 27 respectively, Fig. 7J, L). These data suggest that the neural crest genes are differentially regulated by histone3 methylation.

## Discussion

Neurocristopathies are a group of diverse disorders resulting from defective growth, differentiation, and migration of the neural crest cells. Folate deficiency has long known to be related to developmental neural defects, especially neural tube defects and neurocristopathies [7]–[9], [35], [36]. In mouse the folate receptor Folr1 is expressed at high levels in the dorsal neural tube during neural crest formation [37]. The migration of cardiac neural crest cells are affected in the *Folr1* mutant embryos [38]. In chick embryos, interference with Folr1 expression also reduced the formation and migration of cardiac neural crest cells [39]. We showed here in *Xenopus* embryos that inhibition of RFC blocked the expression of a series of neural crest marker genes including *Zic1*, *Snail1*, *Snail2*, *FoxD3* and *Twist1*, but had no clear effects on *Pax3* and *Msx1* (Fig. 2). At tadpole stages, the XRFC-MO injected embryos developed characteristic neurocristopathies including craniofacial malformation, hypopigmention, heart defects, and gut coiling malformations (Fig. 3).

Folate levels affect DNA synthesis, amino acid metabolism, and methylation reactions via S-adenosylmethionine (SAM) mediated one-carbon transfer reactions. Folate promotes the remethylation of homocysteine, which is cytotoxic and can induce DNA strand breakage, oxidative stress and apoptosis. Low folate induced hyperhomocysteinemia has been suggested to be involved in the developmental defects of the cardiac neural crest in chick embryos [14], [15]. In the XRFC-MO injected embryos, however, no clear change in cell apoptosis or cell proliferation were observed as compared to the control embryos (data not shown). We suggest that a basic level of folate absorption might be maintained by other folate receptors to support the proliferation of the neural crest precursors. Indeed, the classical folate receptor *Folbp1* is widely expressed in the neural plate during neurula stages (data not shown). Instead, our data supported a role of RFC in the epigenetic regulation of neural crest development. First, neural crest development is sensitive to levels of folate and related molecules. Overexpression of *RFC* or injection of 5-MTHF, VB12 or SAM all induced expansion of the expression domains of neural crest genes, while injection of the folate antagonist MTX had an opposite effect (Figs. S2, S3). Second, the neural crest defects in XRFC morphants were largely rescued by co-injection of 5-MTHF, VB12 or SAM. And the function of RFC depended on its activity in folate transportation (Fig. 5). Third, modulating folate levels by knockdown of XRFC or addition of 5-MTHF clearly affected histone methylation levels in *Xenopus* animal caps (Fig. 7), supporting the idea that endogenous folate regulates neural crest gene expression via an epigenetic mechanism. Indeed, bioinformatic analyses suggested that many of the neural crest genes are subjected to epigenetic regulations. Moreover, the neural crest defects in XRFC morphants could be partially rescued by overexpression of the lysine methyltransferases hMLL1 (Fig. 7). Collectively, our data strongly suggest that RFC mediated folate metabolism regulates neural crest gene expression through epigenetic mechanisms.

Among the neural crest genes sensitive to folate levels, *Zic1* is the most upstream gene in our analysis. *Zic1* is also down regulated in the *RFC* knockout mouse as shown by microarray analysis [40]. Intriguingly, overexpression of RFC also rescued the effect of the dominant negative constructs of several downstream genes. One possibility is that the epigenetic regulation promoted by RFC is involved directly in the regulation of a panel of the neural crest GRN genes. Thus RFC might work in parallel with the canonical neural crest GRN, although we can not rule out the possibility that this phenomenon is resulted from the cross-regulation between the neural crest genes. Interestingly, overexpression of hMLL1 promoted the expression of *Zic1* and *FoxD3*, but not *Snail2* and *Twist1*, suggesting differential epigenetic regulations of the neural crest genes. Epigenetic mechanisms have also been implicated in folate deficiency-related neural tube defect mouse model *Splotch*, which carries a loss of function mutation in *Pax3*. In cells from caudal neural tubes of *Pax3* mutant embryos, the expression of the histone demethylases KDM6B decreases and the cells exhibit increased H3K27 methylation [41]. Interestingly, these phenotypes could also be rescued by addition of exogenous folic acid [41]. Pax3 is a key regulator in neural crest development and the H3K4 level of *Pax3* transcript region is also developmentally regulated [28]. However, the expression of *Pax3* itself was not sensitive to folate levels in our study. These data suggest that different mechanisms are likely involved in the epigenetic regulation of the neural crest genes. There is increasing evidence that chromatin modification plays important roles in vertebrate neural crest development. For example, the CHD (chromodomain helicase DNA-binding domain) member CHD7, an ATP dependent chromatin remodeller, is shown to be essential for neural crest specification and migration [42]. Recently, the histone demethylase JmjD2A has been shown to be required for neural crest induction, which is recruited to the regulatory regions of neural crest genes and thus poises neural crest differentiation [43].

Supplementary folate has been shown to have relatively specific protective effects on neural tube and neural crest cells, suggesting that these cells might be more sensitively regulated by folate-related pathways. In addition to folate, microinjection of SAM or VB12 also affects neural crest development in *Xenopus* embryos (Fig. S2), possibly through promoting methylation reactions and thus epigenetic regulations. These data also manifests the role of nutrients in gene transcriptional regulation through epigenetic modification, highlighting the association of dietary intake with epigenetic modification, as well as diseases.

## Materials and Methods

### Ethics Statement

The care of *Xenopus laevis* (Nasco), *in vitro* fertilization procedure and embryos study were performed according to protocols approved by the Ethics Committee of Kunming Institute of Zoology, Chinese Academy of Sciences (permit number: SYDW-2006-006).

### Microinjection and in situ hybridization

*In vitro* fertilization, embryo culture, whole mount *in situ* hybridization,preparation of mRNA, and microinjection were carried out as described [44]. The sequence of the antisense morpholino oligo (MO) for *XRFC* used was: 5′-ATTCTGTGTCATCTCGGCAGCAACT-3′, which was obtained from Gene Tools (OR). Two *XRFC* alleles were found in the EST database and the targeted sequence is 100% conserved between them. For *in situ* hybridization, the probes for *Msx1*, *Pax3*, *Zic1*, *Snail2*, *FoxD3* and *Twist1* were used as described [6], [45]–[47].

### Plasmids construction

Full-length *Xenopus laevis RFC* coding region was obtained by PCR according to sequence in GenBank (accession no. BC073675) and cloned into pCS2+ vector. An EST clone (IMAGE: 3509422) containing full length hRFC was obtained from Open Biosystems and was subcloned into pCS2+ vector. To prepare the pCS-hRFC R133C and R373C mutation constructs, site-directed mutations were carried out by PCR driven overlap extension [48] using *Pfu* DNA polymerase (Fermentas). The GR fusion constructs for inducible expression of XSnail1, XSnail2, Snail1 N, Snail1 Znf, Snail2 N and Snail2 Znf were prepared as described [45].

### Reverse transcription and polymerase chain reaction (RT-PCR) and animal cap assay

To analyze the temporal expression of XRFC during development, RT-PCR was carried out using whole embryos at different developmental stages. The primers used were: *XRFC*: 5′- CTGGTTCCCATAGCCATCTT-3′ and 5′- TTTGGAGGGATTTGAGGTTT-3′. H4 was used as a loading control. For animal cap assay to analyze the effect of XRFC in neural crest induction, the embryos were injected with *XWnt7b* and *tBR* mRNA with or without XRFC-MO or RFC mRNA at 2-cell stage. The animal caps were cut at stage 9 and cultured till control embryos reached stage 15–17, and processed for RT-PCR. The primers used for PCR were: *Pax3*: 5′-CAGCCGAATTTTGAGGAGCAAAT-3′ and 5′-GGGCAGGTCTGGTTCGGAG TC-3′; *Snail2*: 5′ -TCCCGCACTGAAAATGCCACGATC -3′ and 5′- CCGTCCTAAAGATGAAGGGTATCCTG -3′. The primers for *Msx1*, *Zic1*, *FoxD3* and *Snail1* were used as described [49]–[51].

### Neural crest graft experiment and cartilage staining

The donor embryos were injected with EGFP mRNA in both blastomeres at 2-cell stage and the EGFP-labeled neural crest explants were separated at stages 14–17 and implanted into the corresponding regions of host embryos as described [23]. Cartilage staining was carried out using Alcian blue 8GX according to protocol of Dr. Richard Harland's lab (http://tropicalis.berkeley.edu/home/gene_expression/cartilage-stain/alcian.html).

### Western blot analysis

Embryos were injected with XRFC-MO, XRFC-MO+mRNA, or 5-MTHF in all blasomeres at 2-cell stage and animal caps were cut at stage 9 and cultured till control embryos reached St.15–17. The injected and control animal caps were then harvested and lysed in modified TNE lysis buffer (50 mM Tris-HCl [pH 7.4], 150 mM NaCl, 0.5 mM EDTA, and 0.5% Triton X-100) containing protease inhibitor cocktail (Roche) and processed for Western blot. The antibodies used for monomethyl-, dimethyl- and trimethyl-H3-K4 were from Millipore.

## Supporting Information

Figure S1 **RFC is involved in the neural crest gene regulatory network.** (A, B) The inhibition of Zic1 expression by dominant negative Msx1 can be rescued by XRFC. (C, D) Msx1 rescues the inhibition of Zic1 expression by XRFC-MO. (E–H) The inhibition of Snail2 expression by XRFC-MO can be rescued by Msx1, Pax3 and Zic1 co-expression. (I–N) The inhibition of FoxD3 expression by dominant negative Msx1, Pax3 and Zic1 can be rescued by co-injection of XRFC. (O, P) The inhibition effect on neural crest migration (as indicated by the distribution of the Twist1 positive neural crest cells) of Snail1-Znf can be rescued by co-injection of XRFC. The injected sides are indicated by the red staining of tracing lacZ. (Q) Quantification of the rescue effect of RFC on the expression of Zic1, FoxD3 or Twist1 inhibited by dominant negative Msx1, Pax3, Zic1 and Snail1 and the rescue of the XRFC-MO effect by Msx1, Pax3, and Zic1.
(TIF)

Figure S2 **Components of the folate metabolism pathway regulate neural crest gene expression in** ***Xenopus*** **embryos.** (A–H) Both folate transportation deficient forms of hRFC (R133C/R373C) failed to promote neural crest makers expression (Twist1, FoxD3, Snail2 and Zic1). (I–T) Injection of 5-MTHF (20ng/embryo, I–L), Vitamin B12 (50ng/embryo, M–P) or SAM (10ng/embryo, Q–T) promoted neural crest maker genes expression (Twist1, FoxD3, Snail2 and Zic1). (U) Quantification of the promoting effect on the expression of neural crest genes by hRFC, hRFC-R133C, hRFC-R373C and 5-MTHF, SAM, and Vitamin B12.
(TIF)

Figure S3 **The folate antagonist MTX blocks neural crest induction.** (A–C) Injection of MTX (10ng/embryo) inhibits the expression of Twist1, Snail2 and Zic1 in the injected sides. (D–F) Co-injection 5-MTHF (20ng/embryo) with MTX rescues the effects of MTX on neural crest gene expression. (G) Quantification of the inhibitory effect of MTX on the expression of neural crest genes and its rescue by 5-MTHF.
(TIF)
